# Jatrorrhizine alleviates ulcerative colitis via regulating gut microbiota and NOS2 expression

**DOI:** 10.1186/s13099-022-00514-z

**Published:** 2022-10-21

**Authors:** Jia Ling Zhang, Min Na Zhang, Hong Gang Wang, Xiao Zhong Yang, Cheng Gong Yu

**Affiliations:** 1 Department of Gastroenterology, Gulou School of Clinical Medicine, Nanjing Medical University, Nanjing, Jiangsu Province China; 2 Department of Gastroenterology, The Affiliated Huai’an No 1 People’s Hospital of Nanjing Medical University, Huai’an, Jiangsu Province China

**Keywords:** Jatrorrhizine, Colitis, Gut microbiota, 16s rRNA, Network pharmacology, Transcriptome sequencing

## Abstract

**Background:**

The natural protoberberine jatrorrhizine (JA) is reported to have several medicinal properties and a significant effect on the gut microbiota of mice. The regulation of gut microbiota is generally known to play an important role in the intestinal mucosal immune response to ulcerative colitis (UC). However, whether JA can be used in the treatment of UC is still unclear. Our study aimed to investigate the underlying therapeutic effects and mechanisms of JA in treating colitis.

**Results:**

Compared with the DSS-induced colitis model group, the JA + DSS treated group had more significant improvements in weight loss, disease activity index score, colon length shortening, and pathological inflammation. 16s rRNA sequencing analysis showed that JA treatment protected colitis mice against DSS-induced disturbance of gut microbiota. At the phylum level, reductions in Deferribacteres and Proteobacteria were observed in the JA-treated group; At the genus level, the JA-treated group showed an increased relative abundance of *Akkermansia* and decreased abundance of *Escherichia-Shigella, Desulfovibrio, Mucispirillum*, etc. Network pharmacology was then used to screen out five drug-disease target genes (NOS2, ESR1, CALM1, CALM2, CALM3). Transcriptomics analysis further validated that the NOS2 expression was significantly reduced in colon tissue of JA-administered mice compared with DSS control mice. Additionally, analysis of correlation suggested that NOS2 expression was negatively correlated with the relative abundance of *AKKermansia* and positively correlated with *Desulfovibrio, Rikenella*.

**Conclusion:**

JA alleviates ulcerative colitis via regulating gut microbiota and NOS2 expression.

**Supplementary Information:**

The online version contains supplementary material available at 10.1186/s13099-022-00514-z.

## Introduction

With the increasing incidence and prevalence of inflammatory bowel disease (IBD), IBD has emerged as a global challenge in the 21st century [1]. Ulcerative colitis (UC) is a major clinical entity of IBD with unknown etiology [2]. A large number of studies have indicated that interactions of multiple factors, such as genetic susceptibility, and environmental, and microbial changes may lead to the occurrence and development of UC [3–5]. Current conventional drugs for treating UC include 5-aminosalicylic(5-ASA), corticosteroids, immunosuppressants, and biological agents [6] However, clinical treatment remains challenging because of recurrent symptoms, drug resistance, and related side effects. Additionally, 20–25% of patients have symptoms that are difficult to control with drugs and thus eventually require surgical treatment [7]. Therefore, it is of great significance that we find new, natural, and efficient UC therapeutic drugs.

Jatrorrhizine is an isoquinoline alkaloid isolated and extracted from a variety of plant families, predominantly including Menispermaceae, Ranunculaceae, and the Rutaceae [8]. In recent years, Jatrorrhizine has attracted extensive attention from researchers due to its medicinal value. Several studies have revealed that Jatrorrhizine shows multiple pharmacological activities such as antimicrobial [9], anticancer [10], and anti-diabetic [11] effects. The bioactive metabolites produced by jatrorrhizine also exert pharmacological properties by modulating multiple signaling pathways, such as Wnt/β-catenin [12], and miR-223-3p/HDAC4 [13], NF-κB [14], and PPAR-γ [15]. Additionally, Jatrorrhizine treatment was demonstrated to regulate the abundance of gut microbiota and reverses learning and memory deficits [16]. Specially, Jatrorrhizine treatment increased the relative abundance of the probiotics (*Lactobacillus acidophilusm* and *Bifidobacterium*) [16].

Recently, a popular theory is that gut microbiota dysbiosis plays an essential role in UC occurrence and development [17], and therapeutics that improve gut dysbiosis have shown great potential for UC treatment. Accumulating evidence has indicated that the efficacy of traditional Chinese medicine in the treatment of UC is related to changes in the gut microbiota composition [18, 19]. However, the manner which jatrorrhizine protects against UC is still unclear. In our study, we explored the effects of Jatrorrhizine on gut microbiota in DSS-induced colitis mice and further validated the potential therapeutic target of Jatrorrhizine in UC based on network pharmacology analysis. Collectively, our findings show Jatrorrhizine has the potential to become a novel natural drug for the treatment of UC.

## Materials and methods

### Animals and experimental design

Female C57BL/6(16-22 g) mice aged 6–8 weeks were purchased from the Model Animal Research Center of Nanjing University. Using Female mice because females have a higher model rate and are easier to model induction [20]. All mice were maintained at the Center for Experimental Animal of Huai’an First People’s Hospital. The rearing environment had a temperature of 22 ± 2℃, and a humidity of 45 ± 10%, with a 12 h light/12 h dark cycle. Animal experiments were approved by the Huai’an First Hospital Laboratory Animal Ethics Committee affiliated with Nanjing Medical University. After one week of acclimation, the mice were randomly divided into three groups of Jatrorrhizine (JA)-treated, DSS, and Control. A dose of 60 mg/kg of Jatrorrhizine was dissolved in 0.2ml of sterile saline and administered to mice by oral gavage. The JA-treated group was given Jatrorrhizine every other day for one week, and the drinking water was replaced with 2.5% DSS (molecular weight, 36–50 kDa; MP Biomedicals, LLC, Irvine, CA, United States) in the second week, and continue to give the same dose by gavage every other day. The mice in the DSS group drank water freely in the first week and were replaced with 2.5% DSS in the second week. Control mice drank normal water for two weeks. From the second week, the body weight, stool property, and hematochezia of the mice were recorded daily, and the disease activity index (DAI)was calculated. On the 14th day of the experiment, mice were euthanized and fresh feces and colon tissues were collected for further analysis.

### Disease activity indexes (DAI)

DAI score was graded on a scale of 0–4 based on the following parameters: body weight loss (1, 1–5%; 2, 5–10%; 3, 10–15%; and 4, ≥ 15%), stool consistency (0, normal; 2, loose stools; 4, diarrhea), and blood in the stool (0, no blood seen; 2, apparent blood with stool; 4, grossly bloody stool). Loss of body weight by more than 30% was used as a criterion for humane euthanasia to reduce the pain of the mice. As the DAI score was an indicator of daily assessment and measurement, the researchers were not blinded to the grouping of the experiment.

### Histopathological analysis

The distal colon tissue was obtained and fixed in 4% paraformaldehyde for histopathological analysis. Colon tissue samples were dehydrated in gradient alcohol and embedded in paraffin. Sections (4 μm) were stained with hematoxylin and eosin(H&E). The histological score was calculated according to the degrees of inflammation (0, none; 1, mild; 2, moderate; 3, severe), crypt gland damage (0, normal; 1, basal 1/3 damage; 2, basal 2/3 damage; 3, crypt lost and surface epithelium present; 4 crypt and surface epithelium lost), infiltration of lymphocyte (0, 0%; 1, 10%; 2, 10-25%; 3, 25-50%; 4, > 50% ,*The lymphocyte infiltration was observed at 400× magnification), structure of colon wall (0, none; 1, mucosa; 2, submucosa; 3, transmural). The total histological score was expressed as the sum of four parameter scores [21]. The Histological scoring was performed by two independent pathologists who were blind to the experiment condition.

### Gut microbiota analysis

Gut microbiota composition was determined by 16 S rRNA gene sequencing. The fresh fecal samples in mice were collected, and the microbial community genomic total DNA was extracted by an E.Z.N.A.® soil DNA Kit (Omega Bio-Tek, Norcross, GA, U.S.). The NanoDrop 2000 UV-vis spectrophotometer (Thermo Scientific, Wilmington, USA) was used to check the concentration and purity of DNA. Then, the hypervariable V3-V4 regions of the 16SrRNA sequence were then amplified with primer pairs 338 F (5’-ACTCCTACGGGAGGCAGCAG-3’) and 806R(5’-GGACTACHVGGGTWTCTAAT-3’) using an ABI GeneAmp® 9700 PCR thermocycler (ABI, CA, USA) [22]. The PCR amplification was carried out for 3 min at 95 ℃ for initial denaturation, followed by 27 cycles of denaturing at 95 ℃ for 30 s, annealing at 55 ℃ for 30 s, and extension at 72 ℃for 45 s, and single extension at 72 ℃ for 10 min, and end at 4 ℃. The PCR products were purified by the AxyPrep DNA Gel Extraction Kit (Axygen Biosciences, Union City, CA, USA) according to the manufacturer’s instructions and quantified using Quantus™ Fluorometer (Promega, USA). Sequencing was performed on an Illumina MiSeq PE300 platform (Illumina, San Diego, USA).

### 16 S rRNA gene sequence analysis

Bioinformatics 16 S rRNA gene sequencing reads were demultiplexed and quality-filtered using QIIME open-source bioinformatics pipeline for an initial assessment [23]. Fastq data were quality controlled by Trimmomatic and Pear. The screening criteria are as follows: (i) a sliding window strategy is adopted using Trimmomatic v0.36, the window size is set to 50 bp, the average quality value is 20, the minimum reserved sequence length is 120, and Pearv0.9.6 is used to remove sequences with N; (ii) Flashv1.20 and Pear0.9.6 are used to merge the sequences at both ends according to the overlapping relationship of PE. The minimum overlap is set to 10 bp and the mismatch rate is 0.1 to obtain the Fasta sequence; (iii) The chimera of the Fasta sequence was removed by chime method according to the known database, and the self-alignment (denovo) method was used to remove the unknown database, and the undesired short sequences were removed at the same time. Operational taxonomic units (OTUs) were clustered with a 97% similarity using UPARSE (version 7.1). The taxonomy of each 16 S rRNA gene sequence was analyzed by Ribosomal Database Project (RDP) Classifier algorithm2 against the Silva (SSU128) 16 S rRNA database [24].

### Calculation of Diversity and Richness Index

Relative abundance (%) was used to represent the relative abundance of OUT and species [25]. The alpha diversity indexes were estimated based on the OUT using Chao 1 and Shannon [26]. Chao1 is an index of the bacterial species, which is used to estimate the number of OTUs in the community. The formula for this index is Schao1 = Sobs + n1(n1-1)/2(n2 + 1), where Sobs = number of observed OTUs, n1 = the number of OTUs with only one sequence, and n2 is the number of OTUs with only two sequences. Shannon is one of the indices used to estimate microbial diversity in a sample. Its formula is as follows: H= -Σ(Pi) (ln Pi), where Pi is the proportion of individuals belonging to species i in the sample. The larger the Shannon value, the higher the community diversity.

### Transcriptome analysis

Total RNA was extracted from inflamed colon tissue. Samples about 1 cm long were used for sequencing. Then, we collected colon samples from three randomly chosen animals per group for sequencing. Total RNA from colon tissues was isolated with TRIzol reagent. The concentrations of RNA were assessed using a Nanodrop spectrophotometer (IMPLEN, CA, USA). RNA integrity was determined by Agilent 2100 (Agilent Technologies, CA, USA). The sample libraries were generated using NEBNext UltraTM RNA Library Prep Kit for Illumina (NEB, USA) according to the manufacturer’s recommendations. The library was sequenced using Illumina Hiseq 4000 platform and generated paired-end 150 bp reads. We then used Tophat2(v2.1.0) [27] and Cufflinks (v2.1.1) [28] software to complete alignment and analyze transcripts, and make a quantitative analysis of all genes. DEGs analysis was performed according to the read count obtained in the gene expression level analysis. For samples with biological replicates, differential expression analysis was performed using DESeq (1.10.1) [29]. The resulting P-values of differential expression analysis were controlled for FDR (false discovery rate) using the method of Benjamini and Hochberg. The standard of differential gene screening is generally: q-value < 0.05. Additionally, Gene Ontology (GO) enrichment analysis was performed using GOseq software [30] and based on Wallenius non-central hypergeometric distribution. KEGG pathway enrichment analysis was performed using KOBAS software. Rich factor, q-value, and the number of genes enriched in this pathway were used to measure the degree of KEGG enrichment [31]. The rich factor was the value ratio of the number of DEGs in the pathway to all annotated genes enriched in the pathway [28]. A q-value < 0.05 was considered significant enrichment in the KEGG pathways, where the q-value is the adjusted p-value.

### Network pharmacology analysis

The active ingredients of Jatrorrhizine were searched through the TCMSP database (http://tcmspw.com/tcmsp.php) and the target protein corresponding to Jatrorrhizine was screened with the oral availability OB ≥ 30% as the limiting condition. Next, the name of the target protein was standardized through the Uniprot (http://www.uniprot.org/) database, the species was set as “Homo Sapiens (human)”, and the confirmed target was used as the screening condition to construct a data set of potential targets of jatrorrhizine. With “ulcerative colitis” as the keyword, the related targets of UC were searched in three databases of the drug target database (Drug-bank), disease databases gene-disease association database (DisGeNET), and the human gene database (GeneCards). After eliminating the repeated disease targets, all the target genes of each database were merged. Using R software (version 4.1.2) to draw the Venn diagram of the predicted targets of jatrorrhizine and the disease targets of ulcerative colitis, and obtain the intersection targets of the two as potential targets of jatrorrhizine in the treatment of ulcerative colitis. Then, the potential targets were imported into the STRING database to obtain the high-confidence protein interaction relationships of intersecting target proteins. Finally, the protein interaction network was drawn with the Cytoscape software, and the target with the largest degree value was selected as the core target of jatrorrhizine in the treatment of UC.

### Statistical methods

Statistical significance was evaluated using GraphPad Prism 8.0 software. Analysis of variance was used for comparison between groups, and independent samples t-test was used for comparison between two groups. Results are shown as mean ± standard error. Correlation analysis was performed using Pearson correlation analysis in SPSS version 26(IBM SPSS Statistics). All data were normally distributed. P values < 0.05 were considered statistically significant.

## Results

### JA ameliorates DSS-induced colitis

**Fig. 1:**
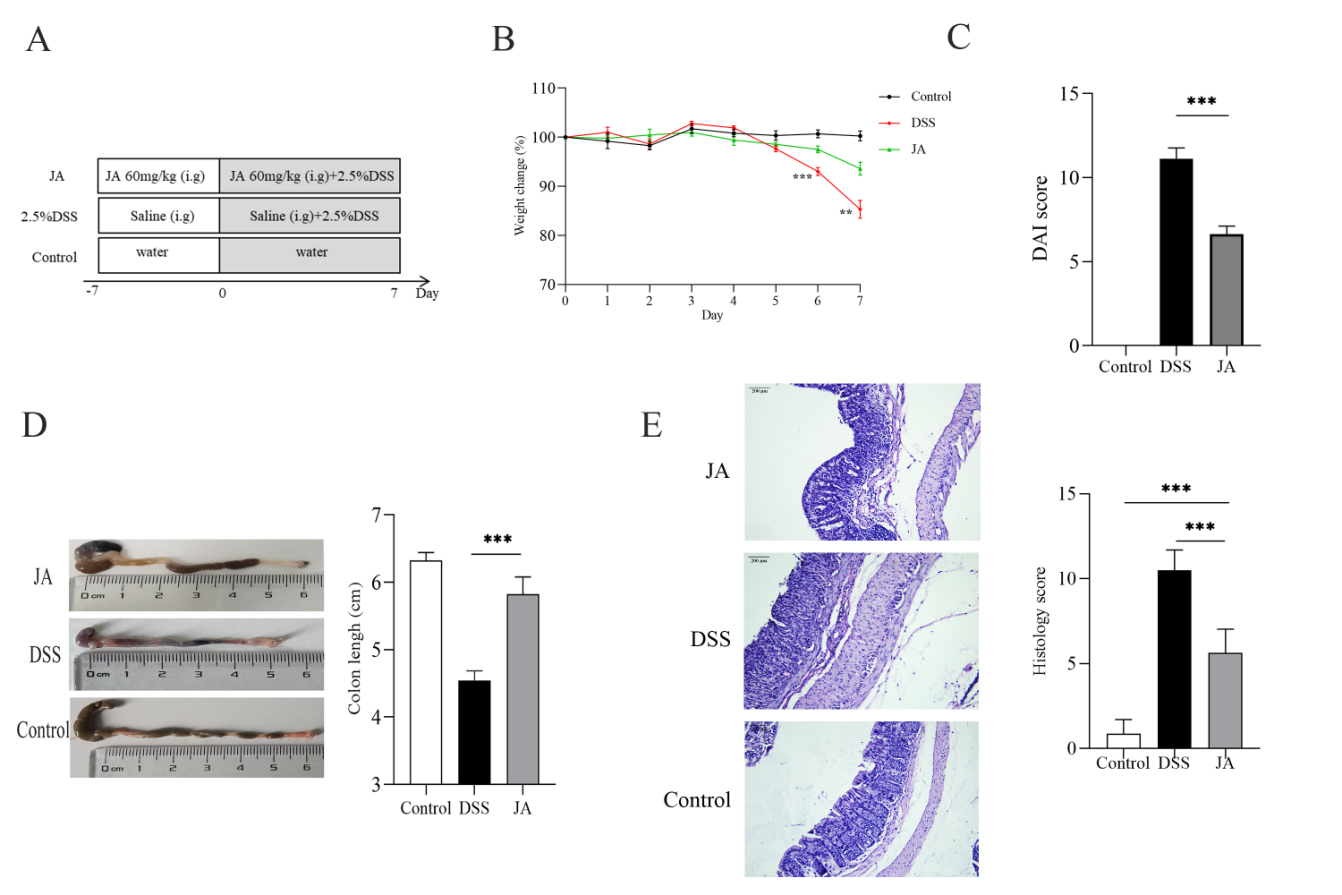
JA ameliorates DSS-induced colitis. (A) Experimental design for the treatment of JA on DSS-induced colitis; (B) Percentage change in body weight; (C) Disease activity index (DAI) value; (D) Colon length; (E) Colon tissue HE staining (50X; 200X); Histology score. Values are presented as the mean ± standard error of the mean (SEM); CON group, n = 8; DSS group, n = 8; JA-treated group, n = 8; *t-test P < 0.05, **t-test P < 0.01, ***t-test P < 0.001

The design of the animal experiments is shown in Fig. 1 A. Twelve female C57BL/10 mice were randomly divided into three groups (n = 8 per group): the Control group, the DSS-induced colitis group, JA-treated DSS-induced colitis group. Mice in the JA-treated group were treated with the Jatrorrhizine by oral gavage for 14 days (from days − 7 to 7), while the control and DSS-only groups were each infused with water and saline, then 2.5% DSS administration to the JA-treated and DSS-only groups started on day 0 of the experiment. As shown in Fig. 1B, compared with the Control group, the body weight of mice in the DSS-only group dramatically decreased after day 6. However, the Jatrorrhizine treatment significantly reduced the weight loss compared with the DSS-only group. The DAI score was used to evaluate the severity of the colitis activity. The DAI score was significantly lower in the JA-treated group than in the colitis group (Fig. 1 C). Shortening of the colon length is a primary indicator of the severity of colonic inflammation [32]. Jatrorrhizine effectively mitigated the colon shortening induced by DSS so that the colons of the JA-treated mice were comparable in length to those of Control mice (Fig. 1D). Additionally, hematoxylin and eosin (HE) staining were used to assess the extent of inflammation in the colonic tissue. Destruction of colonic epithelial cells, distortion of crypt structure, and massive inflammatory cell infiltration were observed in the colon tissues of the DSS group. However, Jatrorrhizine treatment significantly reduced the abovementioned pathological manifestations (Fig. 1E). These results suggested that Jatrorrhizine attenuates DSS-induced colitis.

### JA alters the composition of gut microbiota in colitis mice

**Fig. 2:**
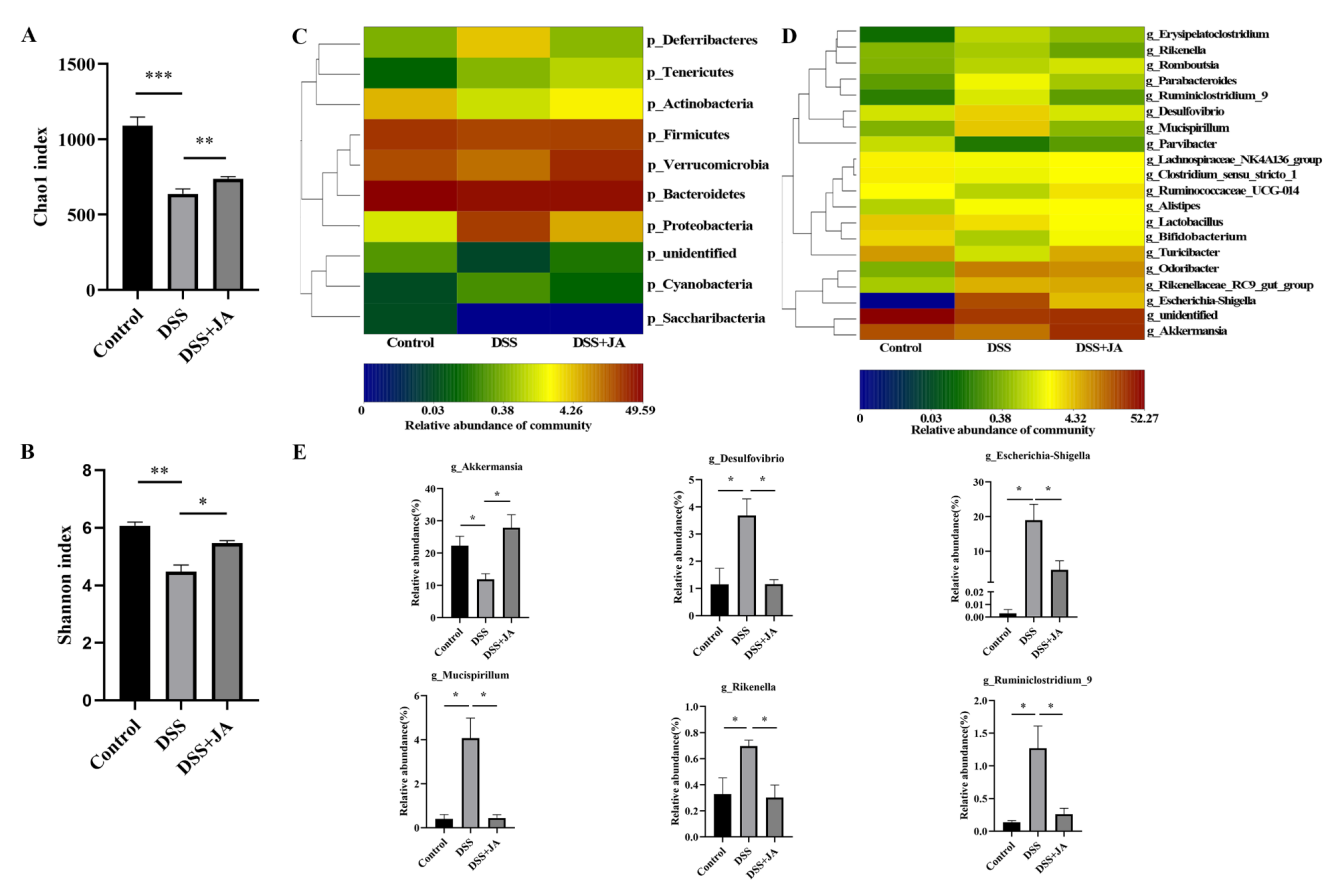
JA alters the composition of gut microbiota in colitis mice. (A) Chao index; (B) Shannon index; (C) Heatmap of gut microbiota at the phylum level; (D) Heatmap of gut microbiota at the genus level; (E) Relative abundance of discriminative gut microbial communities at the genus level; Values are presented as SEM; CON group, n = 3; DSS group, n = 3; JA-treated group, n = 3; *t-test P < 0.05, **t-test P < 0.01, ***t-test P < 0.001

We further explored the effect of Jatrorrhizine gavage on gut microbiota by 16 S rRNA high-throughput sequencing. 393,599 raw sequences from all samples were produced, and a total of 357,383 high-quality sequences were obtained after further removal of chimeras and short sequences. According to the results of operational taxonomic unit (OTU) clustering and used statistical analysis to obtain the Chao1 and Shannon indices, which were used to assess the Alpha diversity of the gut microbiota in the three groups (Fig. 2 A, 2B). The results showed that gut microbiota richness decreased after DSS administration compared with the Control group, while Jatrorrhizine treatment recovered the diversity of gut microbiota to some extent. We subsequently explored in what way the composition of the gut microbiota was affected by Jatrorrhizine. At the phylum level, Verrucomicrobia, Deferribacteres, and Proteobacteria accounted for the majority of the bacteria. Deferribacteres and Proteobacteria were significantly increased in the DSS-only compared with the controls (Fig. 2 C). However, Jatrorrhizine treatment inhibited the effects of DSS on the relative abundance of these phyla in colitis mice. At the genus level, DSS colitis-induction reduced the relative abundance of *Akkermansia* and increased that of *Escherichia-Shigella, Desulfovibrio, Mucispirillum, Ruminiclostridium_9*, and *Rikenella*, while Jatrorrhizine treatment remarkably restored the level of the above microbiome (Fig. 2D and E). Overall, these results indicated that Jatrorrhizine treatment protects against changes to the composition of gut microbiota in DSS-induced colitis mice.

### Effect of Jatrorrhizine on colonic tissue gene expression in mice with UC

**Fig. 3:**
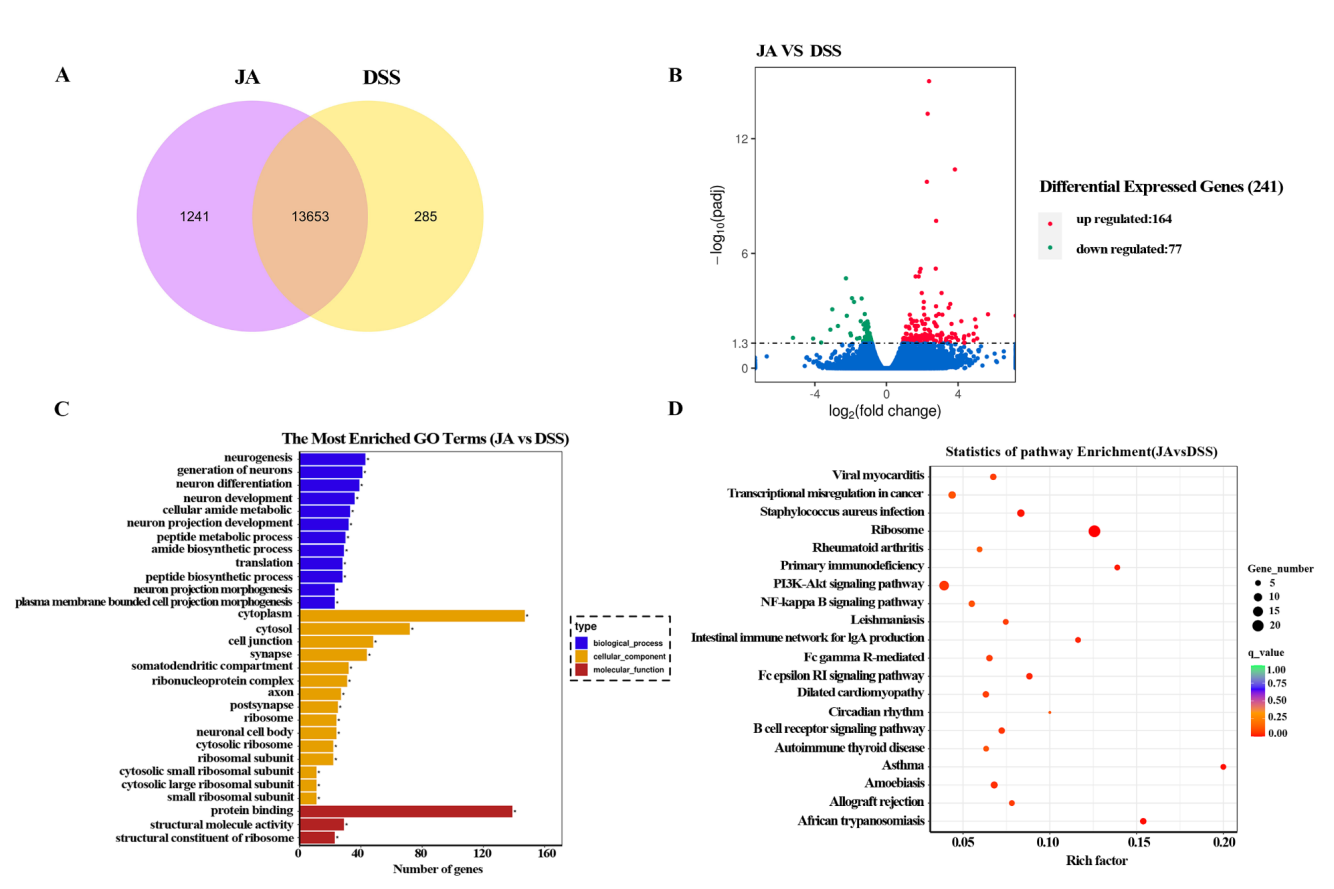
Effects of JA on colonic tissue gene expression in mice with ulcerative colitis. (A) The Venn diagram; (B) Volcano plot analysis identifies DEGs between DSS and DSS + JA group; (C) The most enriched GO terms (JA vs. DSS); (D) The top 20 statistics of Pathway Enrichment

To further investigated the underlying mechanisms of Jatrorrhizine-induced improvement in colonic inflammation, we performed transcriptome sequencing using colon tissue from the JA-treated group and DSS-only group (n=3 per group). Venn graph analysis demonstrated that 13,653 genes were expressed in both groups (Fig. 3 A). While 1241 OTUs were unique to the JA-treated group and 285 OTUs were unique to the DSS group. In addition, the volcano plot in Fig. 3B exhibiting the differentially expressed genes (DEGs) between two groups shows that 164 genes were upregulated and 77 were downregulated after Jatrorrhizine treatment. Next, a functional classification analysis of the DEGs was conducted. DEGs were annotated with GO terms, which were subdivided into three main categories: biological process (BP), molecular function (MF), and cellular component (CC) (Fig. 3 C). The most enriched GO term in the BP category was “neurogenesis”; in the CC category, it was “Cytoplasm”; and in MF, it was “protein binding.” Furthermore, KEGG pathway enrichment analysis was performed to identify potential genes affected by Jatrorrhizine treatment, and the top 20 most significantly enriched pathways are shown in Fig. 3D, these included ribosome, primary immunodeficiency, and intestinal immune network for IgA production. The most notable of these is the ribosome signaling pathway. Additionally, we also listed 28 DEGs according to |log2foldchange|>2 (Supplementary Table 1).

### Potential target of Jatrorrhizine against colitis verified based on network pharmacology

**Fig. 4:**
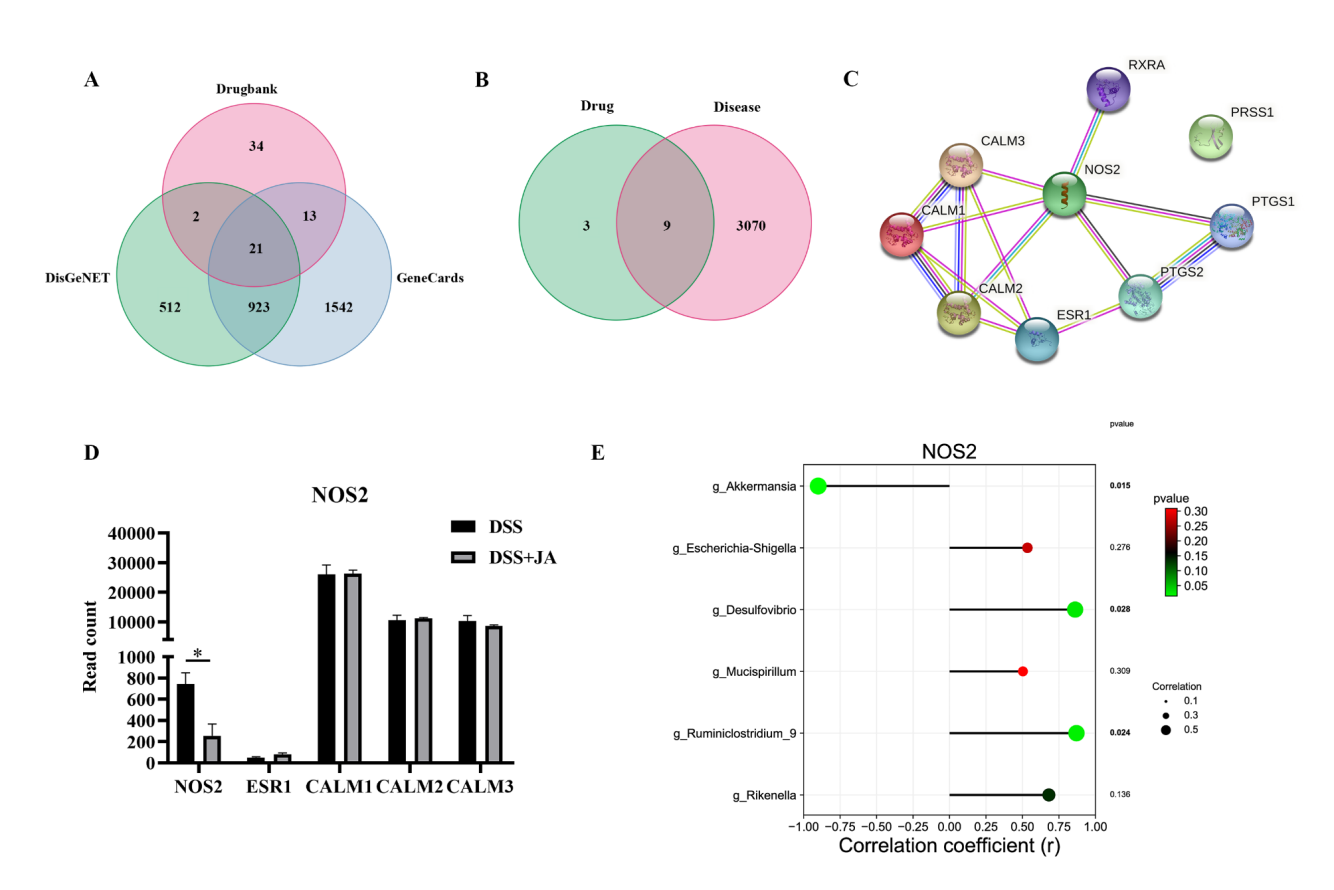
The potential target of Jatrorrhizine against colitis verified based on network pharmacology and transcriptomics analysis. (A) The Venn diagram of the overlapping targets from three databases. (B) The Venn diagram of drug-disease intersection target. (C) Read counts value of target genes were compared between two groups (D) The Pearson correlation analysis between the NOS2 expression and differential gut microflora

In the above experiments, we found Jatrorrhizine to have a positive effect on DSS-induced animal models of colitis. Thus, we hoped to further explore the possible target of Jatrorrhizine in the treatment of UC patients. As shown in Table 1, we obtained 10 potential molecular targets for jatrorrhizine from the TCMSP database. After importing the targets and screening for confirmed genes related to humans using the UniProt database, 12 genes corresponding to JA targets were obtained. Next, we collected 70 UC-related target genes from Drugbank, 1458 target genes from DisGeNET, and 2499 target genes from the GeneCards database (Fig. 4 A). These target genes were combined and de-duplicated to obtain 3079 disease targets. Then, we identified nine common targets based on intersections of 12 jatrorrhizine targets and 3079 UC targets and constructed a Venn diagram using R software (version 4.1.2), which is shown in Fig. 4B. These key targets are RXRA, NOS2, PTGS1, PTGS2, PRSS1, ESR1, CALM1, CALM2, and CALM3. Afterward, the nine drug-disease intersecting co-targets were uploaded to the STRING database to construct a protein-protein interaction (PPI) network (Fig. 4 C) and imported into the Cytoscape software for further analysis. Ultimately, the top five genes were screened according to degree values: NOS2, ESR1, CALM1, CALM2, and CALM3 (Table 2).

**Table 1:** Jatrorrhizine molecular information from TCMSP

MolId	MolName	OB (%)	DL	Target
MOL006397	jatrorrhizine	30.44	0.75	Nitric oxide synthase, inducible
				Prostaglandin G/H synthase 1
				Potassium voltage-gated channel subfamily H member 2
				Estrogen receptor
				Androgen receptor
				Prostaglandin G/H synthase 2
				Retinoic acid receptor RXR-alpha
				Trypsin-1
				Nuclear receptor coactivator 2
				Calmodulin

**Table 2:** Degree of gene target in PPI network

Genesymbol	Degree
NOS2	12
CALM1	8
CALM2	8
CALM3	8
ESR1	8
PTGS2	6
PTGS1	4
RXRA	2

### Correlation analysis of the expression of NOS2 and gut microbiota

We then analyzed the transcriptome data from colons of mice treated with DSS and with or without Jatrorrhizine to validate these five potential drug-disease gene targets. We retrieved the read counts values in the transcriptome data of the above target gene, and performed a preliminary screening verification by t-test. As shown in Fig. 4D, NOS2 gene expression was significantly decreased in the Jatrorrhizine-treated compared to the untreated DSS-induced colitis (P < 0.05). Additionally, based on the above results, Jatrorrhizine treatment significantly protected the diversity and structure of the gut microbiota. We wished to further explore whether there was a correlation between gut microbiota and NOS2 gene expression. Correlation analysis revealed that NOS2 gene expression was negatively correlated with the relative abundance of *AKKermansia* (P < 0.05) and positively correlated with *Desulfovibrio*, and *Rikenella*. Taken together, these results provide potential molecular evidence for Jatrorrhizine changing the gut microbiota to treat ulcerative colitis.

## Discussion

Several studies have shown evidence that gut dysbiosis is closely related to UC [33, 34]. Together, decreased intestinal barrier function, an imbalance in microbiota homeostasis, and a lack of beneficial microbial products lead to the destruction of intestinal epithelial cells and abnormal immune responses, which are important factors in the development of UC [35]. In our study, Jatrorrhizine was found to significantly alleviate intestinal inflammation in DSS-induced colitis mice by reducing body weight loss, colon length shortening, DAI score, and histological score. Additionally, based on high-throughput sequencing, Jatrorrhizine was confirmed to protect against the disturbance of the gut microbiota caused by DSS to a certain extent Then, when we combined network pharmacology and transcriptomics to further explore possible targets of jatrorrhizine in the treatment of UC. We screened out the key target NOS2 and found its expression to have a certain correlation with changes in the relative abundance of several gut microbiota. Our study revealed that Jatrorrhizine has the potential to be a therapeutic drug for ameliorating intestinal inflammation in UC by modulating the gut microbiota.

It is well known that interactions between the host and enteric microbes maintain gut homeostasis, help the host resist pathogenic attacks, and promote host immunity and health [36]. The available evidence indicates that reduced gut microbiota diversity is the most consistent indicator of UC [37]. In this study, we found that jatrorrhizine treatment significantly increased the gut microbiota diversity in mice with colitis. In terms of the gut microbiota composition, Jatrorrhizine treatment decreased the abundance of Deferribacteres and Proteobacteria at the phylum level. A study has shown that the increased abundance of Deferribacteres is closely related to the increased formation of neutrophil extracellular traps [38]. Moreover, several studies have found that an increased abundance of Deferribacteres has a pathogenic role in DSS-induced colitis [39, 40]. Moreover, it has been suggested that an expansion of Proteobacteria is representational of gut dysbiosis [41], which is consistent with a previous study that showed the abundance of Proteobacteria was increased in IBD patients [42, 43]. At the genus level, Jatrorrhizine treatment increased the relative abundance of beneficial bacteria *Akkermansia*, while reducing the relative abundance of pathogenic bacteria including *Escherichia-Shigella* and *Desulfovibrio*. *Akkermansia* was confirmed to play an essential role in maintaining barrier function, host metabolism, and immune response. Macchione et al. showed that supplementation with *Akkermansia* provides a protective effect in DSS-induced mouse colitis [44]. Thus, *Akkermansia* was proposed as a potential probiotic. Conversely, *Escherichia-Shigella* was verified to be a potential risk factor [45]. Increased levels of *Escherichia-Shigella* in the inflamed colonic mucosa from UC patients positively correlated with the disease severity [46]. Similarly, *Desulfovibrio* was a hydrogen sulfide-producing opportunistic pathogen, which can damage intestinal epithelial cells and increase intestinal permeability [47]. Collectively, Jatrorrhizine has a certain regulatory effect on the disturbance of gut microbiota in DSS-induced mice.

We further explored the possible mechanisms of jatrorrhizine in alleviating colitis from two different directions. We first constructed drug-disease targets using a network pharmacology method, and then screened out the key targets NOS2, ESR1, CALM1, CALM2, and CALM3 based on a topological analysis of the PPI network. We then performed transcriptome sequencing analysis of mouse colon tissue, and the results suggested that Jatrorrhizine treatment may affect ribosome signaling pathways. We subsequently verified the mRNA expression of the above five targets in colon tissue and found that the expression level of the NOS2 gene dramatically decreased after Jatrorrhizine treatment (P < 0.05).

Ribosomal proteins, as an important part of ribosomes, have been reported to be involved in the balancing of immune function [48]. For example, the mTOR/S6K1 signaling pathway is closely related to cytokine regulation and plays an important role in regulating intestinal immune homeostasis and inflammation response [49]. The intervention of this pathway promises to be a new mechanism for the treatment of UC. S6K1 is a typical ribosomal protein kinase. Phosphorylated S6K1 could accelerate the synthesis and growth of mTOR protein. It can be seen that the synthesis status of ribosomal proteins is closely related to the progression of UC disease. Additionally, a study has shown that inducible nitric oxide synthase (iNOS) encoded by NOS2 plays an important role in inflammatory diseases [50]. Nitric oxide synthase (NOS) isozymes are divided into three subtypes according to their origin: nNOS, iNOS, and endothelial NOS. NOS is a critical enzyme for NO generation, and the production of NO can be indirectly determined in vivo by detecting NOS levels. However, it is still controversial whether NO exerts a pro-inflammatory effect or anti-inflammatory effect. At present, scholars believe that this is closely related to the amount of NO, and small amounts of NO generated by constitutive NOS are considered to be protective, while large amounts of NO are considered to be pro-inflammatory and detrimental [51]. Several studies have confirmed that the activity and expression of iNOS are significantly increased in colonic mucosa in patients with UC [52, 53]. However, the mechanism of the cell damage caused by iNOS is not entirely clear. One theory is that the excess NO interacts with superoxide anions to generate peroxynitrite, which can react with tyrosine in the protein to form nitrate. The results of Immunohistochemical confirmed that positive staining for nitrotyrosine is indeed seen in the inflamed intestinal mucosa of UC patients [54]. Correspondingly, Bernstein H et al. showed that inhibition of NOS2 could alleviate intestinal inflammation, suggesting that the high expression of NOS2 is of great significance in the intestinal inflammatory response with UC [55]. In conclusion, the activation of the ribosomal pathway and the high expression of NOS2 is closely related to UC disease activity.

Interestingly, NOS2 was not only one of the differentially expressed genes for Jatrorrhizine-treated ulcerative colitis, but its expression level was negatively correlated with the relative abundance of *Akkermansia*. A study published by Byndloss et al. showed that the metabolite butyrate of gut microflora can attenuate the expression of NOS2 in colonic epithelial cells and suppress the synthesis of iNOS by activating the PPAR-γ signaling pathway. Simultaneously decreased NO production in the colon and decreased nitrate levels in the intestinal lumen, and the nitrate is a specific source of the proliferation of enteropathogenic bacteria [56]. Similarly, Lukovac et al. also found that the metabolites of *Akkermansia* including propionate and butyrate significantly reduce the expression of the PPAR-γ gene [57]. Therefore, a consideration of all the evidence above suggests that altered gut microbiota abundances probably related to NOS2 expression.

## Conclusion

Collectively, our study results showed, for the first time, that jatrorrhizine alleviates DSS-induced colitis via regulating the composition of gut microbiota in colitis mice, affecting the ribosome signaling pathway, and reducing the expression of the NOS2 gene. Therefore, we speculated that the alleviating effect of jatrorrhizine on colitis may be related to the metabolic regulation and function of the gut microbiota. Collectively, these results provide a basis for further research aimed toward the mechanism by which Jatrorrhizine treats UC by regulating the gut microbiota.

## Electronic supplementary material

Below is the link to the electronic supplementary material.

Supplementary Material 1

## Data Availability

The datasets used and analyzed during the current study are available from the corresponding author on reasonable request.
