# Supplementary material for: Jatrorrhizine alleviates ulcerative colitis via regulating gut microbiota and NOS2 expression
Source: Gut Pathog. 2022 Oct 21;14:41. doi: 10.1186/s13099-022-00514-z (PMC9587631; doi:10.1186/s13099-022-00514-z)
Supplement: Supplementary file 1 — Supplementary Material 1 [file 13099_2022_514_MOESM1_ESM.docx]

**Supplementary Table 1 The DEGs between the JA and DSS group**

| **Gene_id** | **readcount_JA** | **readcount_DSS** | **log2FoldChange** | **padj** | **Gene name** |
| --- | --- | --- | --- | --- | --- |
| ENSMUSG00000052305 | 282.516906 | 2477.351442 | -3.1324 | 0.0097532 | Hbb-bs |
| ENSMUSG00000095633 | 8.139002068 | 66.32223364 | -3.0266 | 0.00084378 | Igkv4-58 |
| ENSMUSG00000076583 | 20.12953251 | 131.6547583 | -2.7094 | 0.0062832 | Igkv8-24 |
| ENSMUSG00000105606 | 71.68396171 | 333.1446968 | -2.2164 | 0.0018304 | Igkv2-109 |
| ENSMUSG00000099398 | 86.27311553 | 21.13134575 | 2.0295 | 0.004837 | Ms4a14 |
| ENSMUSG00000026764 | 86.10894447 | 20.4705034 | 2.0726 | 0.000713 | Kif5c |
| ENSMUSG00000023885 | 205.040467 | 48.31720427 | 2.0853 | 0.000345 | Thbs2 |
| ENSMUSG00000027438 | 99.18716577 | 22.93724447 | 2.1125 | 0.001613 | Napb |
| ENSMUSG00000021567 | 65.37800341 | 14.91488557 | 2.1321 | 0.009194 | Nkd2 |
| ENSMUSG00000021087 | 100.6702385 | 22.65808758 | 2.1515 | 0.004837 | Rtn1 |
| ENSMUSG00000055407 | 52.96604177 | 11.36618471 | 2.2203 | 0.002732 | Map6 |
| ENSMUSG00000039278 | 135.1731827 | 26.86359189 | 2.3311 | 0.002732 | Pcsk1n |
| ENSMUSG00000054162 | 46.22456692 | 7.28063314 | 2.6665 | 0.004037 | Spock3 |
| ENSMUSG00000021303 | 58.46094499 | 8.798934519 | 2.7321 | 0.004981 | Gng4 |
| ENSMUSG00000032181 | 63.30059518 | 9.296171142 | 2.7675 | 0.006283 | Scg3 |
| ENSMUSG00000027318 | 101.1814864 | 14.83014382 | 2.7703 | 0.000584 | Adam33 |
| ENSMUSG00000027581 | 70.33803973 | 10.26459921 | 2.7766 | 0.00183 | Stmn3 |
| ENSMUSG00000062760 | 58.02576977 | 8.260064352 | 2.8125 | 0.006518 | Shisal1 |
| ENSMUSG00000031137 | 42.71553058 | 5.647323832 | 2.9191 | 0.001491 | Fgf13 |
| ENSMUSG00000029838 | 207.705632 | 24.60392742 | 3.0776 | 0.000118 | Ptn |
| ENSMUSG00000032532 | 39.25486393 | 4.510707912 | 3.1214 | 0.001613 | Cck |
| ENSMUSG00000050711 | 160.9648064 | 14.46808407 | 3.4758 | 0.0007 | Scg2 |
| ENSMUSG00000035864 | 76.05074359 | 6.42995779 | 3.5641 | 0.000442 | Syt1 |
| ENSMUSG00000046844 | 81.8376792 | 6.600165084 | 3.6322 | 0.004864 | Vat1l |
| ENSMUSG00000024907 | 66.41194203 | 3.666453576 | 4.179 | 0.003509 | Gal |
| ENSMUSG00000055567 | 32.37418357 | 1.046929369 | 4.9506 | 0.002802 | Unc80 |
| ENSMUSG00000034098 | 18.18665655 | 0.5671169 | 5.0031 | 0.006946 | Fstl5 |
| ENSMUSG00000037428 | 19.43372256 | 0.38168546 | 5.67 | 0.001531 | Vgf |
